# Usability of a Co-designed eHealth Prototype for Caregivers: Combination Study of Three Frameworks

**DOI:** 10.2196/26532

**Published:** 2021-08-18

**Authors:** Melanie Tremblay, Karine Latulippe, Manon Guay, Véronique Provencher, Anick Giguère, Valérie Poulin, Véronique Dubé, Dominique Giroux

**Affiliations:** 1 Department of Teaching and Learning Studies Université Laval Québec, QC Canada; 2 School of Rehabilitation Université de Sherbrooke Sherbrooke, QC Canada; 3 Center of Research on Aging Centre intégré universitaire de santé et de services sociaux de l’Estrie Centre hospitalier universitaire de Sherbrooke Sherbrooke, QC Canada; 4 Center of Excellence on Aging Quebec Québec, QC Canada; 5 Department of Family Medicine and Emergency Medicine Université Laval Québec, QC Canada; 6 Université du Québec in Trois-Rivières Trois-Rivières, QC Canada; 7 Interdisciplinary Center for Research in Rehabilitation and Social Integration Université Laval Québec, QC Canada; 8 Research Centre of the University Hospital of Montreal Montreal, QC Canada; 9 School of Social Work and Criminology Université Laval Québec, QC Canada; 10 Department of Rehabilitation Université Laval Québec, QC Canada

**Keywords:** usability evaluation, co-design, research methods, caregivers, service providers, product objectives

## Abstract

**Background:**

Co-design (or the participation of users) has shown great potential in the eHealth domain, demonstrating positive results. Nevertheless, the co-design approach cannot guarantee the usability of the system designed, and usability assessment is a complex analysis to perform, as evaluation criteria will differ depending on the usability framework (or set of criteria) used. ISO (International Organization for Standardization) on usability (ISO 9241-210), Nielsen heuristic, and Garrett element of user experience inform different yet complementary aspects of usability.

**Objective:**

This study aims to assess the usability and user experience of a co-design prototype by combining 3 complementary frameworks.

**Methods:**

To help caregivers provide care for functionally impaired older people, an eHealth tool was co-designed with caregivers, health and social service professionals, and community workers assisting caregivers. The prototype was a website that aims to support the help-seeking process for caregivers (finding resources) and allow service providers to advertise their services (offering resources). We chose an exploratory study method to assess usability in terms of each objective. The first step was to assess users’ first impressions of the website. The second was a task scenario with a think-aloud protocol. The final step was a semistructured interview. All steps were performed individually (with a moderator) in a single session. The data were analyzed using 3 frameworks.

**Results:**

A total of 10 participants were recruited, 5 for each objective of the website. We were able to identify several usability problems, most of which were located in the *information design* and *interface design* dimensions (Garrett framework). Problems in both dimensions were mainly coded as *effectiveness* and *efficiency* (ISO framework) and *error prevention* and *match between the system* *and the real world* (Nielsen heuristic).

**Conclusions:**

Our study provided a novel contribution about usability analysis by combining the 3 different models to classify the problems found. This combination provided a holistic understanding of the usability improvements needed. It can also be used to analyze other eHealth products.

**International Registered Report Identifier (IRRID):**

RR2-10.2196/11634

## Introduction

### Background

eHealth is becoming increasingly important to support people in taking care of their own health and that of their loved ones. In 2019, more than 90% of Canadians had access to the internet and 50% reported having access to at least one web-based health service [1]. The COVID-19 pandemic has amplified its use. eHealth is notably one of the solutions that can support caregivers in the daily tasks required to care for an older person at home [2]. Guay et al [3] suggest that internet-based interventions can have positive effects on the psychological well-being of caregivers of older persons*.* Irani et al [4] reported that people with chronic diseases and their caregivers were satisfied with the use of the technology. However, some faced technical challenges, whereas others were concerned about the technology’s lack of a personalized approach. Moreover, caregivers of functionally dependent older persons are often older themselves. In 2012, in Québec (Canada), 41% of caregivers were aged over 55 years [5]. A digital divide related to age and education [6], which are both determining factors of internet use [7], still remains. Concerns can, therefore, be raised about the acceptability of eHealth solutions within this group of users, as many factors influence older people’s acceptance of technology, such as privacy implications and usability factors [8].

In response to this issue, there is a growing interest in the co-design approach [9]. In this approach, researchers, designers, and participants are cocreating with users, who are considered experts of their experience and play a large role in knowledge development, idea generation, and concept development [10]. Authors have reported that the participation of different actors in a co-design project allowed a better understanding of each other’s perspective and reality [9,11]. As co-designers, caregivers and older adults can share their concerns and expectations about the technology in a democratic process, which might increase the fit between their needs and the system developed. However, the co-design approach cannot guarantee the usability and user experience (UX) of the designed system.

### Usability and UX Evaluation

#### Usability Definition

Usability is the “functional relationships between people and the products and systems they use” [12]. It is also defined as the “extent to which a system, product or service can be used by specified users to achieve specified goals with effectiveness, efficiency and satisfaction in a specified context of use” [13]. Users do not want a difficult or uncomfortable experience in their interaction with the system, but usability requires more than just the users’ desire for it [14]. To achieve usability of the system, we need to evaluate it and adjust the design to address the problems found. Usability assessment is a complex analysis to perform, as evaluation criteria will differ depending on the usability framework (or set of criteria) used.

#### The International Organization for Standardization Framework

The ISO (International Organization for Standardization) usability framework (ISO 9241-210) is a framework accepted worldwide [13] to assess usability in general. The criteria of this framework are all part of the ISO’s definition of usability: *effectiveness*, *efficiency*, *satisfaction,* and *context of use*. The *specified users,* *specified goals*, and *context of use* are a combination of the situated aspects of the interaction with the system: who are the users, what do they want to achieve with the system, and in which context (at home or at work, on their phone or on their computer, etc)? All these factors need to be considered when assessing the usability of a system. *Effectiveness* is the *accuracy and completeness with which users achieve specified goals*. Are users able to achieve the task? *Efficiency* refers to the *resources used in relation to the results achieved*. How long and how easy was it to accomplish the task? *Satisfaction* is defined as the “extent to which the user’s physical, cognitive and emotional responses that result from the use of a system, product or service meet the user’s needs and expectations” [13]. Did users appreciate their interaction with the system while performing the task?

This framework allows a general picture of usability but cannot provide specific insights into what is needed to achieve better results. How can we fix a problem related to effectiveness? The Nielsen heuristic framework (1995) provides more details on what the system should do to meet the ISO criteria.

#### The Nielsen 10 Usability Heuristics Framework

Heuristics describe an approach to problem solving whereby people will rely on a limited number of principles to reduce the complexity of a task by *predicting values to simpler judgmental operations* [15]. Heuristics are helpful in predicting the reaction of users interacting with a system. The Nielsen framework (1995) listed 10 heuristics to consider while assessing or trying to achieve usability:

1. *Visibility of system status:* “The design should always keep users informed about what is going on, through appropriate feedback within a reasonable amount of time.”
2. *Match between the system and the real world:* “The design should speak the user’s language. Use words, phrases, and concepts familiar to the user, rather than internal jargon. Follow real-world conventions, making information appear in a natural and logical order.”
3. *User control and freedom:* “Users often perform actions by mistake. They need a clearly marked ‘emergency exit’ to leave the unwanted action without having to go through an extended process.”
4. *Consistency and standards:* “Users should not have to wonder whether different words, situations, or actions mean the same thing. [Words, situations, and actions should] follow platform and industry conventions.”
5. *Error prevention:* “Good error messages are important, but the best designs carefully prevent problems from occurring in the first place. Either eliminate error-prone conditions or check for them, and present users with a confirmation option before they commit to the action.”
6. *Recognition rather than recall:* “Minimize the user’s memory load by making elements, actions, and options visible. The user should not have to remember information from one part of the interface to another. Information required to use the design (e.g. field labels or menu items) should be visible or easily retrievable when needed.”
7. *Flexibility and efficiency of use:* “Shortcuts, hidden from novice users, may speed up the interaction for the expert user, such that the design can cater to both inexperienced and experienced users. [The system should] allow users to tailor frequent actions.”
8. *Esthetic and minimalist design:* “Interfaces should not contain information which is irrelevant or rarely needed. Every extra unit of information in an interface competes with the relevant units of information and diminishes their relative visibility.”
9. *Help users recognize, diagnose, and recover from errors:* “Error messages should be expressed in plain language (no error codes), precisely indicate the problem, and constructively suggest a solution.”
10. *Help and documentation:* “It’s best if the system doesn’t need any additional explanation. However, it may be necessary to provide documentation to help users understand how to complete their tasks [16].”

Refer to the NNGroup website [16] for a detailed description, with examples of each heuristic. These heuristics are guidelines for achieving *effectiveness, efficiency,* and *satisfaction*. They focus on the task goals. However, users’ goals are not always task oriented (do-goals) [17]. Other goals, such as be-goals, will affect their experience with technology [18]. Users are not just users. They are also human beings with feelings. The UX shifts the focus from the product to feelings while users interact with the technology [19].

#### Garrett Framework: The Elements of UX

The elements of UX framework [20] proposes 5 dimensions to describe UX design: strategy, scope, structure, skeleton, and surface. Each dimension has distinctive elements (Textbox 1).

Each framework provides a different understanding of how a product can get closer to what users really want. These frameworks inform different yet complementary aspects of usability and UX.

**The elements of user experience and their description.**

**Dimension, Elements, and Description**

- Strategy
  Product objectives: what are the business goals or other specific goals the product is aiming for?
  User needs: who are the target users and what do they want?
- Scope
  Functional specifications: what functionalities are required to address user needs and product objectives?
  Content requirements: what content is required to address user needs and product objectives?
- Structure
  Interaction design: how does the system behave in response to the users’ actions?
  Information architecture: what is the structural arrangement and distribution of information throughout the system?
- Skeleton
  Information design: how is the information presented to facilitate understanding?
  Interface design: how are the interface elements organized on the page to enable users to interact with the system?
  Navigation design: what elements allow the user to access the different sections of the information architecture?
- Surface
  Sensory design: what sensory (vision, touch, etc) experience is created by the product?

### Objective

The objective of this study is to assess the usability and UX of an early version co-designed prototype to support the help-seeking process of caregivers of functionally dependent older persons. Trying to gather as much information as possible on potential improvements, we want to explore the contribution of the 3 frameworks presented: the ISO [13], Nielsen usability heuristics [16], and Garrett elements of UX [20].

## Methods

### Context of the Study

**Figure 1:**
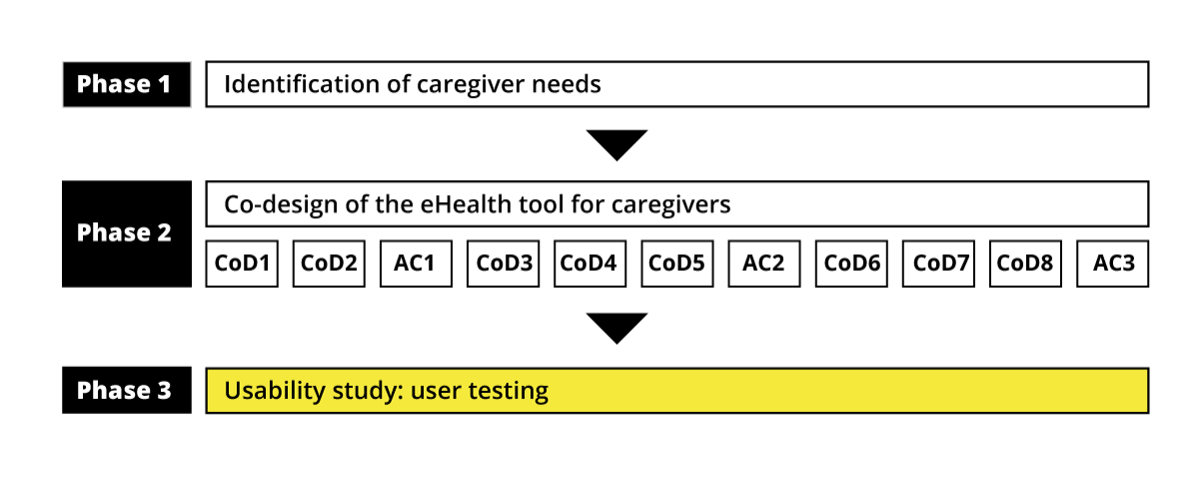
Context of the study. Ac: advisory committee session; CoD: co-design session.

On the basis of the potential value of the co-design approach in the eHealth domain, we first co-designed an eHealth prototype to support the help-seeking process of caregivers of functionally dependent older persons (Figure 1). The co-design phase of the study (phase 2) included 8 co-design sessions and 3 advisory committee meetings held in 11 of the 16 administrative regions of the province of Québec from May 2017 to June 2018.

The research project protocol of the entire study can be found in the paper by Latulippe et al [21], and results on user needs, requirements, and overall process and design decisions are presented in 3 papers [22-24]. A total of 74 co-designers were recruited, including 30 caregivers, 26 community workers, and 18 health professionals. Advisory committee meetings were held in plenary and co-design sessions were held in both plenary and subgroup workshops and included different types of activities depending on the objectives of the session.

The eHealth prototype developed was a website with 2 main objectives: helping caregivers to find resources (with a search tool and a questionnaire to help identify the needs) and allowing service providers to offer their services. The prototype is currently hosted on a private server.

### Explorative Usability and UX Assessment

We chose an exploratory study method to assess usability, as the prototype was in its first version [25,26]. Changes were made to phase 3 of the initial protocol to gather more appropriate knowledge about usability and UX, considering the state of the prototype [21]. These changes include the addition of users’ first impressions, the accuracy of the methods used to perform the think-aloud method, and the use of a semistructured interview rather than a standardized questionnaire.

### Recruitment

Participant recruitment included recruiting for the 2 objectives of the website (offering and finding resources for caregivers). Two researchers (KL and MC) completed the recruitment and data collection. All participants were recruited from a single region of Québec for feasibility reasons. The first inclusion criterion was potential users of the website. We contacted service providers via telephone and email. Service providers helped to recruit caregivers within their organization. One inclusion criterion for service providers was to provide services to caregivers of functionally dependent older persons. One inclusion criterion for caregivers was to provide assistance on a regular basis (at least once a week) to a person aged 65 years or older. Participating in phase 2 (co-design of the tool) was not an inclusion criterion for recruitment in the usability study (phase 3), but it was also not an exclusion criterion. As the objective of this study was exploratory and we were testing with an early version of the prototype, we targeted 10 users, including service providers and caregivers. We wanted an equal representation of participants for each objective of the website. Faulkner [27] revealed that the average percentage of problem areas found in 100 trials of 5 users found 85% (38/45), ranging from 55% (25/45) to nearly 100% (45/45), whereas groups of 10 found 95% (43/45) of the problems.

### Data Collection

#### Global Process

**Figure 2:**
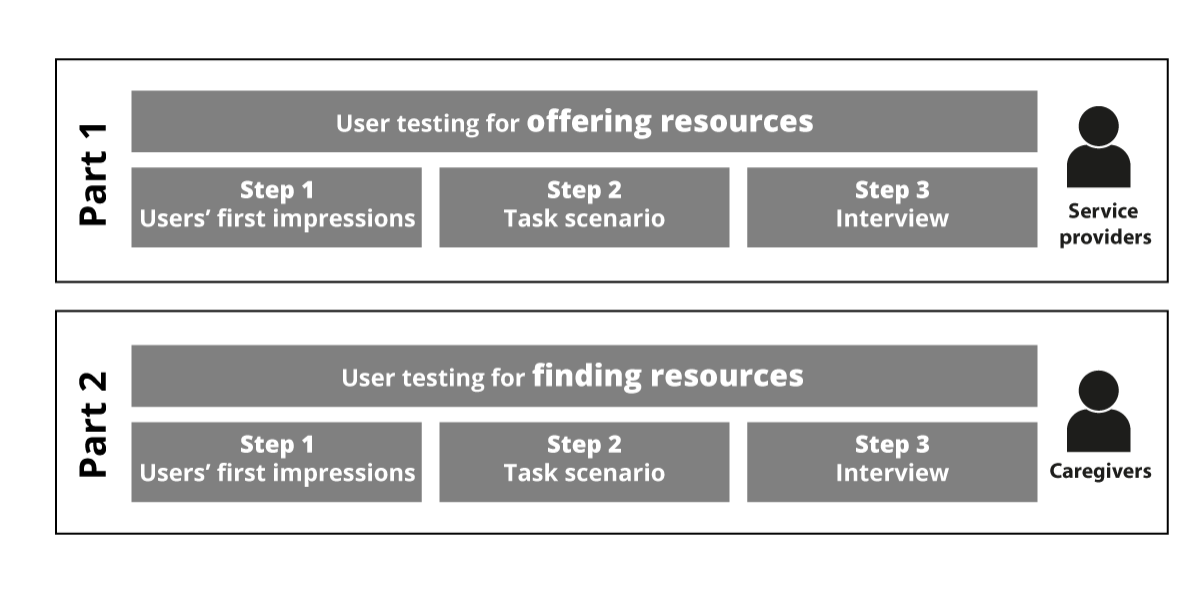
Study methodology.

As the prototype was in the early stages of development, the database did not contain any resources except the *test* resource entered by researchers during the programming of the prototype. Thus, we completed data collection in 2 parts through individual user testing, including 3 steps each (Figure 2). For the first part, we evaluated usability and UX for the *offering resources* objective. Participants contributed to adding some resources in the database in which caregivers would eventually search. For the second part, we evaluated usability and UX for the *finding resources* objective with caregivers. We collected data in French, the main language used in Québec.

We completed the 3 steps of each part in a single session of 30-45 minutes with each participant. All sessions were videotaped. We conducted tests at the workplace for service providers and at home (or in a community center) for caregivers. Moderators used either a portable PC with a webcam and the Open Broadcast Studio (The OBS Project) [28] or the participant’s own computer, a camera or an iPad, and an audio recorder. Open Broadcast Studio is a free open-source software that allows the recording of multiple sources of data simultaneously. The webcam captures the participants’ reaction. The integrated audio captor records participants’ verbalization, and the desktop or browser windows are captured as another source.

#### Step 1: Users’ First Impressions

We used the 5-second test (5ST) to gather the first impression of users. This involved a display of the home page for 5 seconds, followed by questions [29,30]. The 5ST technique was used to gather information about general eye-catching attributes of the home page and provided general first impressions. After the 5 second display, we asked participant, “Can you tell me what you remember seeing?” To collect more detailed information about the perception of the utility for each user subgroup, we added a second display of the home page without a time limit. Participants were then asked to express their perceived usefulness, “As a [caregiver or service provider], what do you think you could do with this website?”

#### Step 2: Task Scenarios

The second step included task scenarios [25] with a coaching think-aloud protocol [31]. The task scenarios represented several tasks that the user would typically perform with this website and put them in context [25]. Scenarios differed depending on the targeted subgroup. Each scenario included a practice task to familiarize participants with the think-aloud protocol, followed by 5 assessed tasks. We selected the tasks based on what each subgroup of users would typically want to do with the website and on specific interactions that the research team wanted to assess (Textbox 2). In accordance with the *coaching think-aloud* protocol, moderators worked with participants during task performance. When the participant stopped talking during the task, the moderator repeated the instruction, *keep thinking aloud, please*. When a participant struggled with a task, the moderator provided some guidance.

**Task scenarios and related questions.**

**Part 1: Offering Resources**

- Find resources
  “You want to find resources for a caregiver you interact with. How would you proceed?”
- Create a profile
  “You want to create a profile for your organization. How would you proceed? Please create a complete profile.”
- Add an activity
  “You want to add an activity offered by your organization. How would you proceed?”
- Add a document
  “You want to add an information document presenting details of your organization’s services. How would you proceed?”

**Part 2: Finding Resources**

- Identifying their own needs
  “You are tired and you need help but you do not know exactly what you are looking for. What would you do?”
- Finding a resource in their region
  “You want to find a support group in your region. How would you proceed?”
- Adding a search result to their favorites
  “You want to keep the name of an organization to go back to it more quickly. How would you proceed?”
- Finding a document
  “You want a document suggesting strategies for bathing assistance. How would you proceed?”

#### Step 3: Semistructured Interview

We created an interview guide based on validated usability questionnaires [32-35]. We created our own interview guide because validated questionnaires have limited applicability and are not suited to all systems [36]. We also wanted to address the specific objectives of the usability evaluation and UX of our prototype, such as problems faced during task performance [25]. We included 8 questions, with probing questions adapted to our designed prototype in the interview guide to answer more specifically to our study objectives (Textbox 3).

**Questions for the semistructured interview.**

**Questions**

1. “When you were [task], I noticed [negative attitude, discomfort, difficulties, time to perform tasks]. Can you tell me why you had [negative attitude, discomfort, difficulties, time to perform tasks]?”
2. “Is the website easy to use?”
  *Probing question:* “What seems complicated to you?”
3. “Is the organization of the website logical and optimal?”
  *Probing question:* “What is inconsistent in the website’s organization?”
4. “Did you find information easily?”
  *Probing question:* “Which information did you not find easily?”
5. “When navigating on the website, is it easy to know where you are?”
  *Probing question:* “When were you not able to know where you were?”
6. “Generally speaking, are you satisfied with this website?”
7. “Do you feel comfortable using this website?”
8. “Would you like to use this website for your tasks?”

To collect sociodemographic data, participants were asked questions about their age, profession, education level, and profession. We also asked 3 multiple-choice questions to assess participants’ perceptions of their technology profiles (Textbox 4).

**Sociodemographic data collection.**

**Multiple-Choice Questions and Their Answer Choices**

- Frequency of internet use
  Several times a day
  Several times a week
  About once a week
  About once a month
  Never
- Ability to find information on the internet
  Always
  Most of the time
  Occasionally
  Rarely
- Comfort level with technology in general
  Rate from 1 to 10 (1 being very uncomfortable and 10 being very comfortable)

### Data Analysis

One researcher (corresponding author) performed the data analysis. We conducted qualitative data analysis in Microsoft Excel using the video recordings of each session. For the first (user impression) and third (semistructured interviews) steps, we conducted an inductive thematic analysis [37]. All participant verbalizations (answers) were transcribed, and some answers were translated by the author for publication purposes. We numbered each answer and collected a list of 5 data items for each (Textbox 5). We used the filter functionality to group and analyze the data.

For the second step (task scenarios), we conducted a deductive analysis [37] based on each criterion of the 3 frameworks [13,16,20]. We entered participant observations and verbalizations for each task. We numbered each problem found during the tasks and registered details for 12 items, each being a column in the Microsoft Excel spreadsheet. Textbox 5 presents a list of the items collected for each step.

For step 2, we first coded each problem according to one criterion of each framework. We then combined the coding for all frameworks using the pivot table functionality. We selected the Garrett [20] criteria to organize the identified heuristic [16] of each problem in the rows field and crossed them with ISO categories [13] in the columns field in the Microsoft Excel spreadsheet.

The study received ethical approval from the *Comité d’éthique de la recherche sectoriel santé des populations et première ligne* (2016-2017-10 MP). Informed consent was obtained from each participant, who also received a nominal compensation of Can $20 (US $16.45).

**List of data items collected.**

**Items Collected at Each Step**

- Step 1 (first impression) and step 3 (semistructured interview)
  Data input number
  Participant ID
  Participant category (service provider or caregiver)
  Source of data (question)
  Data (transcript)
- Step 2 (task scenarios)
  Data input number
  Participant ID
  Participant category (service provider or caregiver)
  Source of data (observation, verbalization, or both)
  Data (transcript or description of observation)
  Problem identified
  Task
  Source of error
  Potential solution
  International Organization for Standardization category
  Heuristic category
  Garrett category

## Results

### Participants’ Demographics

We recruited a total of 10 participants: 4 caregivers and 6 service providers. We conducted 5 user tests for each phase (part 1: offering resources and part 2: finding resources; Table 1). One service provider participated as if she were a caregiver (phase 2). She represented what could happen in a real-context setting: a service provider helping a caregiver.

**Table 1:** Participants’ sociodemographic data (N=10).

Sociodemographic items		Offering resources (n=5)	Finding resources (n=5)
**Gender, n (%)**			
	Women	3 (60)	4 (80)
	Men	2 (40)	1 (20)
Age (years), mean (SD; range)		53.4 (13.4; 42-75)	71.2 (7.8; 58-78)
**Education level, n (%)**			
	College	2 (40)	N/A^a^
	Bachelor’s degree	1 (20)	4 (80)
	Master’s degree	1 (20)	N/A
	Doctorate	1 (20)	1 (20)
**Frequency of internet use, n (%)**			
	Several times a day	1 (20)	1 (20)
	Several times a week	3 (60)	4 (80)
	About once a week	1 (20)	0 (0)
**Capacity to find information on the internet, n (%)**			
	Always	1 (20)	2 (40)
	Most of the time	4 (80)	3 (60)
Comfort level with technology in general from 1 to 10, mean (SD; range)		7.9 (1.02; 6-9)	7 (0.70; 6-8)

^a^N/A: not applicable.

### Step 1: Users’ First Impressions

Table 2 presents the results (emerging categories) for the 5ST of the home page. Elements most commonly identified by participants were located in the top-right section of the page (n=10).

**Table 2:** Participants’ first impressions of the home page (5-second test; n=10).

Interface section			Caregivers mentions (n=5), n (%)		Service providers mentions (n=5), n (%)
**Header (9 mentions)**					
	The phone number of the helpline for caregivers	1 (20)		3 (60)	
	The name of the website	0 (0)		1 (20)	
	The log-in (to access the user profile)	1 (20)		2 (40)	
	Caregivers support (logo)	0 (0)		1 (20)	
**Top left (7 mentions)**					
	A search tool	2 (40)		2 (40)	
	Finding resources	2 (40)		1 (20)	
**Top right (10 mentions)**					
	The definition of a caregiver	3 (60)		0 (0)	
	The question, “are you a caregiver for an elderly person?”	2 (40)		2 (40)	
	Examples of what is a caregiver	0 (0)		2 (40)	
	Caregiver of an older person	0 (0)		1 (20)	

**Figure 3:**
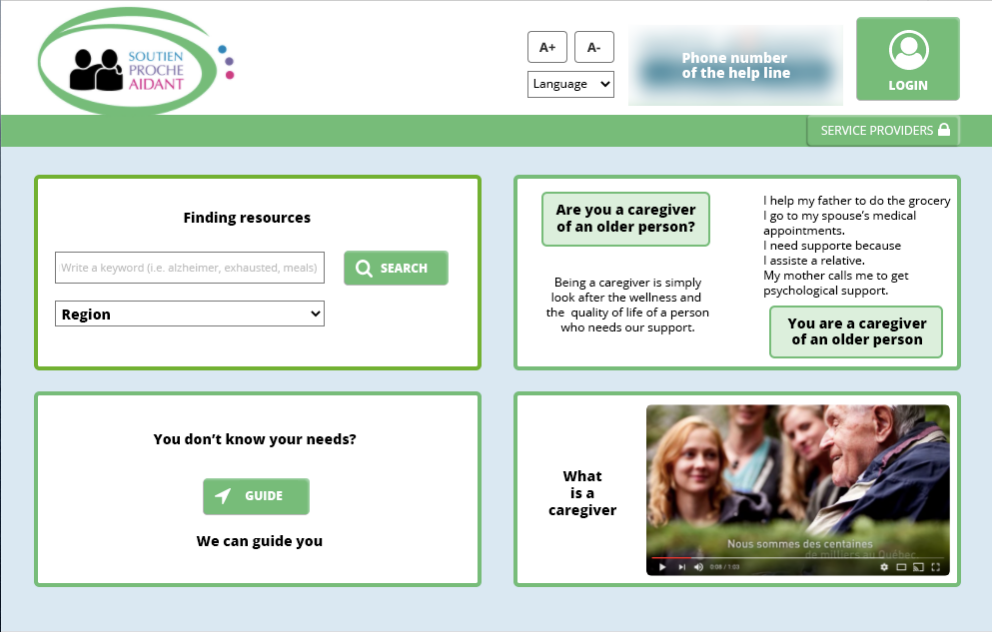
Home page display.

The top left section, where the search tool is located, was among the elements that were perceived less frequently. It was perceived by 7 participants, with only 4 participants mentioning the search tool, which corresponds to the *finding resources objective*. Figure 3 shows the home page displayed to the participants.

After the 5ST, for the second home page display (without a time limit), all service providers answered that they thought the website was for *finding resources to refer or help caregivers*. One service provider also mentioned the requirement of registering its organization. All caregivers answered that they could search or find resources or service providers; 2 of them insisted on the fact that they could find respite resources.

### Step 2: Task Scenarios

#### Summary

We identified 151 problems with the task scenarios, which were classified according to the categories of the 3 frameworks. Some participants were not able to perform all the tasks because of programming errors. When problems were programming errors, they were classified as N/A (not applicable) in each category. This was also done if the focus on the task seemed to interfere with the participant’s perception, for example, if the participant did not see an item they were asked about (the phone number of the helpline for caregivers) because the focus was not on that item but on the task (finding a resource in their region). In those cases, we did not classify the problems, as the task could have affected the interpretation of a category. Most problems (49/151, 32.5%) occurred during the creation of a profile for service providers. The other tasks with a considerable number of problems for service providers were *finding resources* (18/151, 11.9%) and *adding an activity* (10/151, 6.6%). For caregivers, 11.3% (17/151) of problems occurred during the task *finding a resource in their region* and 8.6% (13/151) of problems during the task *identifying their own needs*. The following sections provide a description of these problems.

#### ISO Classification

Table 3 presents findings related to the ISO usability framework. The table presents the number of problems in each category, an example for each with data input number, participant ID, and the transcript or description of the observation. Service providers are identified as SP# and caregivers as C#. It also presents the task where the problems occurred and the type of problem.

**Table 3:** Problems with the website, classified according to the International Organization for Standardization framework.

Usability criteria (ISO^a^ 9241-210)		Problems, n (%)	Task (example)	Verbalization or observation (example)	Problem detected (example)
**Effectiveness**			Finding a resource in their region	“So, I will go to Find resources, I will search for the region first” [#23, C1]	The participant writes nothing in the search bar before clicking on the search button
	Total (n=151)	68 (45)			
	SP^b^ (n=68)	50 (73.5)			
	Caregiver (n=68)	18 (26.5)			
**Efficiency**			Creating a profile	“It is really annoying to have to scroll. I don’t know if I can just write it, how does it work? it is not simple.” [#6, SP1]	Too much scrolling to choose the city
	Total (n=151)	49 (32.5)			
	SP (n=49)	31 (63.3)			
	Caregiver (n=49)	18 (36.7)			
**Satisfaction**			Finding a resource in their region	Participant is using the advanced research functionality; “No, it isn’t the right solution, there are no options!” [#83, C2]	The participant is looking for the word *activity* in the advanced research
	Total (n=151)	18 (11.9)			
	SP (n=18)	9 (50)			
	Caregiver (n=18)	9 (50)			
**Context of use**			Creating a profile	Participant is clicking on the log-in (the one for the caregiver) and then clicks on creating a profile. The participant receives an error message. [#88, SP4]	The participant did not see the access dedicated to service providers
	Total (n=151)	4 (2.6)			
	SP (n=4)	4 (100)			
N/A^c^ (n=151)		12 (7.9)	Question asked after finding a resource in their region	“Did you see the phone number for the help line?” [#21, C1, interviewer]; “I was focusing on the resource, I would have seen it after.” [participant]	The participant did not see the phone number (focus on the task)

^a^ISO: International Organization for Standardization.

^b^SP: service provider.

^c^N/A: not applicable.

Almost half of the problems (68/151, 45%) were classified as *effectiveness* and occurred mainly for service providers during part 1 (50/68, 74%). A total of 32.5% (49/151) were classified as *efficiency*, again mostly by service providers (31/49, 63%). A few problems (18/151, 11.9%) were related to *satisfaction*. Four problems were classified as *context of use*. These problems were identified by a single participant, and most of them (3/4, 75%) could be explained by the fact that this participant was visually impaired. The problems coded as *context of use* included the following: (1) the participant tried to connect or create a profile using the caregiver access instead of the service provider access, (2) insufficient color contrast, (3) and the size of user interface elements.

#### Nielsen Heuristic Classification

Regarding the classification using Nielsen heuristics, *match between the system and the real world* was the most important heuristic principle identified, representing 19.9% (30/151) of problems (Table 4). This heuristic was mostly identified for problems arising for service providers (21/30, 70%) when performing the c*reate a profile* task (12/30, 40%). Other frequently mentioned usability principles included *help and documentation* (25/151, 16.6%), *user control and freedom* (22/151, 14.6%), *error prevention* (21/151, 13.9%), and *recognition rather than recall* (18/151, 11.9%), all of which were again mostly for service providers during the *create a profile* task.

**Table 4:** Classification of problems according to Nielsen heuristics.

Heuristic principle		Problems, n (%)	Task (example)	Verbalization or observation (example)	Problem detected (example)
**Match between the system and the real world**			Create a profile	At the end of the task, the interviewer is pointing on the screen to the access for service providers. The participant says, “It’s locked!” [#108, SP5]	The lock icon is perceived as an item locked on the screen
	Total (n=151)	30 (19.9)			
	SP^a^ (n=30)	21 (70)			
	Caregiver (n=30)	9 (30)			
**Help and documentation**			Create a profile	“What is my username? What was asked before to connect, my email address?” [#7, SP1]	The participant enters their email in the field for the website instead of the email field
	Total (n=151)	25 (16.6)			
	SP (n=25)	19 (76)			
	Caregiver (n=25)	6 (24)			
**User control and freedom**			Create a profile	“I have a video of a caregiver online on my website.” [#16, SP1]	There is no option to add a hyperlink to a video, only to upload one
	Total (n=151)	22 (14.6)			
	SP (n=22)	13 (59.1)			
	Caregiver (n=22)	9 (40.9)			
**Error prevention**			Create a profile	The participant clicks on the log-in instead of the create a profile button (#48, SP3)	The create a profile button is beneath the connection button
	Total (n=151)	21 (13.9)			
	SP (n=21)	14 (66.7)			
	Caregiver (n=21)	7 (33.3)			
**Recognition rather than recall**			Add a research result in their favorites	“I could add it in the favourite of my browser...Here I don’t know what to do” [#135, C3]	The favorite button is not appearing when the user is not connected
	Total (n=151)	18 (11.9)			
	SP (n=18)	11 (61.1)			
	Caregiver (n=18)	7 (38.9)			
**Visibility of system status**			Find a resource in their region	She is clicking on the search button and nothing seems to happen (#147, C4)	The system is not providing information about the action performed
	Total (n=151)	8 (5.3)			
	SP (n=8)	5 (62.5)			
	Caregiver (n=8)	3 (37.5)			
**Esthetic and minimalist design**			Find resources	“And here you have the XYZ Volunteer Center...Three times!” [#25, SP2]	The same research result is appearing 3 times
	Total (n=151)	6 (3.9)			
	SP (n=6)	3 (50)			
	Caregiver (n=6)	3 (50)			
**Help users recognize, diagnose, and recover from errors**			Add a document	An error message appears: “I assume it is because I did not upload a document?” [#39, SP2]	The upload of a document failed
	Total (n=151)	4 (2.6)			
	SP (n=4)	3 (75)			
	Caregiver (n=4)	1 (25)			
**Consistency and standards**			Create a profile	The participant did not enter the postal code or the region of the city. (#53, SP3)	The input fields for the postal code and region are located beside the other input field, on the right side, rather than below them
	Total (n=151)	2 (1.3)			
	SP (n=2)	2 (100)			
**Flexibility and efficiency of use**			Add an activity	“If the activity added appears below, it is a bit annoying. We don’t know if it worked or not.” [#36, SP2]	The added activity is located under the field for adding an activity
	Total (n=151)	2 (1.3)			
	SP (n=2)	2 (100)			
N/A^b^ (n=151)		13 (8.6)	Find a resource in their region	The participant clicks on support group, but nothing happens. (#139, C3)	Link is not working (programming error)

^a^SP: service provider.

^b^N/A: not applicable.

#### Garrett Elements of UX Classification

Table 5 presents the results of the analysis using the Garrett framework. Most of the problems (113/151, 75.8%) were classified in the *skeleton plane*, especially in the *interface design* (54/151, 35.8%) and *information design* (50/151, 33.1%) dimensions. For *interface design*, 65% (35/54) of problems occurred for service providers, mostly to *create a profile* (20/54, 37%). For *information design*, 80% (40/50) of problems occurred for service providers, mostly to *create a profile* (24/50, 48%). Only one problem was categorized in the *user needs* category, and it was also classified for *context of use* in the ISO categorization. This problem reflects an accessibility problem for visually impaired users, meaning that the prototype did not address the specific needs of visually impaired users.

**Table 5:** Classification of problems with Garrett elements of user experience.

Plane and user experience element			Problems, n (%)	Task (example)	Verbalization or observation (example)	Problem detected (example)
**Strategy**						
	Product objectives		0 (0)	N/A^a^	N/A	N/A
	**User needs**			Finding resources	Even if the user is not able to see the entire page at once, she is still able to find the item on the screen, such as the search button. (#86, SP4)	The website is not offering options for visually impaired people
		Total (n=151)	1 (0.7)			
		SP^b^ (n=1)	1 (100)			
**Scope**						
	Functional requirements		0 (0)	N/A	N/A	N/A
	Content requirements		0 (0)	N/A	N/A	N/A
**Structure**						
	**Interaction design**			Finding a resource in their region	“I can ask a question, can’t I?” [#82, C2]	The participant would like to ask a question instead of using the search engine
		Total (n=151)	19 (12.5)			
		SP (n=19)	11 (57.9)			
		Caregiver (n=19)	8 (42.1)			
	Information architecture		0 (0)	N/A	N/A	N/A
**Skeleton**						
	**Information design**			Creating a profile	“What does 24-h surveillance mean? It’s not clear.” [#112, SP5]	The wording is not understood
		Total (n=151)	50 (33.1)			
		SP (n=50)	40 (80)			
		Caregiver (n=50)	10 (20)			
	**Interface design**			Finding a resource in their region	The participant clicks on the description of a support group and nothing happens. (#77, C2)	Only the title is clickable
		Total (n=151)	54 (35.8)			
		SP (n=54)	35 (64.8)			
		Caregiver (n=54)	19 (35.2)			
	**Navigation design**			Finding a resource in their region	The participant and the interviewer are retyping the initial website address to return to the home page. (#65, C1)	The return to the home page with the logo is not understood
		Total (n=151)	9 (6)			
		SP (n=9)	3 (33.3)			
		Caregiver (n=9)	6 (66.7)			
**Surface**						
	**Sensory design**			Finding a document	“What we are trying to do is to colour code the organization in blue, the activities in pink, and the document in purple. You didn’t notice the colour coding?” [#84, C2, interviewer]; “Not at all! I don’t see the point.” [C2]	The participant did not notice the change of color depending on the type of result
		Total (n=151)	6 (3.9)			
		SP (n=6)	4 (66.7)			
		Caregiver (n=6)	2 (33.3)			
**N/A**						
	No classification (n=151)		12 (7.9)	Finding a resource in their region	The participant is entering information in the advance research engine without looking at the results first. (#129, C3)	Due to a programming error, results are not showing besides the advance research box, but below it

^a^N/A: not applicable.

^b^SP: service provider.

#### Combining the Frameworks

Combining all 3 frameworks of analysis provides a comprehensive picture of the identified problems. Multimedia Appendix 1 presents the problems in terms of the dimension of the Garrett framework and the category of ISO usability criteria. The combination also identifies the Nielsen heuristic usability guideline the problem does not address. This table indicates that for *interface design,* problems were mainly identified for *effectiveness* (22/54, 41%) and efficiency (24/54, 44%), with several problems of *error prevention* (ie, when trying to connect to their profile). Problems are also found for *effectiveness* (19/50, 38%) and *efficiency* (25/50, 50%) in *information design,* concerning especially the *match between the system* *and the real world*, mostly for service providers during the creation of their profile and the *help and documentation* (eg, caregiver was looking for the word *Respite* and did not think of entering it in the search engine). The combination of frameworks allows a better understanding of usability problems and provides greater insight into the improvements needed. The numbers in the cells indicate the number of problems at the intersection of the row and the column.

### Step 3: Semistructured Interview

#### Overview

This section presents the questions and translations of the transcripts, including answers to each question. Service providers are identified as SP [#] and caregivers as C [#]. Question 1 was asked during task performance, and the results were included at that point.

#### Question 2

The second question was as follows: “Is the website easy to use?”—Seven participants answered this question. Answers varied among participants: 3 answered “yes” (SP4, C1, and C5), 1 specifying that it was easy to understand and that the screen was not overloaded (C1). Two answers seemed ambivalent:

We find resources when it [website] works properly. If I am looking for an organization but I don’t know the organizations...Finding resources, I don’t know the resources, it is not clear.

SP1

Middle. Knowing if it [the search] worked or not. But visually it’s quite easy, not overloaded. It is easy to search.

C3

One participant answered negatively:

It makes me feel incompetent. I can’t immediately find what I’m looking for.

SP5

#### Question 3

The third question was: “Is the organization of the website logical and optimal?”—Seven participants answered this question. Two were positive:

Personally, I think it’s OK. I will sit with the caregiver and find resources.

C5

Oh yes! We have a lot to learn. If I had it, I would learn a lot!

C4

Three participants said it was clear, but not optimal (SP4, SP5, and C3). Similarly, 2 participants mentioned it could be better (SP2), 1 commenting on the information: “For me, information needs to be precise, I don’t want to get lost in things that will take time” [C2].

#### Question 4

Next, the following question was asked to six participants: “Did you find information easily?”—Only 1 answer was fully positive: “Yes, indeed. It should have info for each organization” [C1].

Other answers were more mitigated: “Yes and no. For now, there is not a lot in it” [SP4], with some commenting on the fact that they needed the interviewer to complete the task (C3 and C4). Two were negative:

Not really. I was not able to get results.

SP2

It was too long.

C2

One participant commented on the information he was not able to find easily:

I expected to arrive directly in the activities, because now, I have to go through the list before getting to the activities. Especially because it is presented...In the list, the organizations were first, then the activities and after the documents. There should be some logic to it.

C1

#### Question 5

The fifth question was as follows: “When navigating on the website, is it easy to know where you are?”—All participants who answered this question (n=8) said they were mostly able to figure out where they were. Three participants answered “yes” (SP2, SP4, and C5); one answered “Quite easily” [C2]; and another answered “Yes, I think” [C4]. Other answers were ambivalent, with participants providing some explanation:

The first time no, but after yes.

C3

It’s because we could not see the rest. I knew where I was in the section I could reach.

SP1

I knew where I was in the website. But, when I clicked here [browser Back button], I expected to go back to the page I was before, but it brings me back to the beginning.

C1

#### Question 6

The following question was answered by only 1 participant: “Generally speaking, are you satisfied with this website?”

More or less. Contrasts should be adjusted for older and colour-blind people.

SP4

#### Question 7

The following question was: **“**Do you feel comfortable using this website?”—Two of those who answered this question (n=4) answered “yes” (SP4 and C3). One specified that she would use it with a digital tablet (C2). Another (SP2) mentioned that he would be somewhat comfortable using it, even with the current problems. He was referring to one of the programming problems.

#### Question 8

For the last question: “Would you like to use this website for your tasks?”—Again, 2 (of a total of 3) participants answered simply “yes” (SP4 and C3). One participant indicated that he would use it with caregivers:

Of course, I would use it! I would use it with the caregiver to help him develop his ability to find information with this tool.

SP6

## Discussion

### Principal Findings

This study aims to assess the usability and UX of the 2 objectives of an early co-designed prototype. Findings from step 1 (users’ first impressions) indicate that participants were able to identify the 2 main objectives of the website. Moreover, even if participants were ambivalent regarding information retrieval, (answers from question 3 during step 3) and the ease of use of the website (answers for question 2 during step 3), they were still comfortable and interested in using the website (answers from questions 5 to 7 during step 3). On the other hand, results from the task scenarios (step 2) tend to indicate that there were more usability problems for the *offering resources* objective, especially when service providers were trying to create their profile. However, this was not the website’s main objective, as the co-design study first aimed to conceive an eHealth tool to support the help-seeking process of caregivers. The second objective (*offering resources*) emerged early during the co-design process, that is, during the identification of functional and content requirements [23]. As mentioned by Luck [38], in participatory design research, knowledge is constructed through practice, and one cannot entirely foresee the direction of the experiment. This was the case, for example, for the co-design study by Tironi [39], in which new knowledge about the ontological perspective of users forced the redefinition of the initial protocol.

### Required Improvements on Accessibility

A second finding relates to accessibility. Accessibility “means removing barriers that might prevent someone from using something, regardless of their access needs” [40]. Accessibility problems were found during task scenarios (step 2) for 1 service provider. Due to a visual impairment, this participant was unable to see the entire page at once. The participant was using a special device to enlarge the interface on the screen. Even if it is uncommon to identify *context of use* as a usability category (other studies generally use the effectiveness, efficiency, satisfaction triad), we chose to include it in our study to see whether we would be able to classify problems in that category, and we were able to do so. The special needs of this participant were not addressed. We recognize that no participant with a visual impairment was included during the co-design process [22]. To maximize the potential of addressing all user needs, participants with special needs should have been included in the co-design process. As mentioned by Cahill [41], co-design or participatory action research is precisely an appropriate method for *including excluded perspectives* and challenging typical knowledge production. From a social justice perspective, other co-design studies should include users with impairments, as special techniques to co-design with them are offered in the literature [42,43].

### Combination of Frameworks

The combination of the 3 frameworks was a novel contribution. It has broadened the perspective and enhanced the strength of our study. As pointed out by Lacerda and von Wangenheim [44] in their systematic literature review, current usability models have many problems (lack of information on the intended use, data collection instruments, and assessment process), leading researchers to seek other sources or combine different models and methods. In our study, the use of the ISO framework was particularly helpful in revealing an important accessibility issue. The use of the Garrett framework was decisive in identifying the dimensions in which the problem was located. Nielsen heuristics helped us to understand how to improve the website in further iterations of the prototype. Each framework provided useful insights to understand the usability and UX of our prototype. However, the combination of the analyses of all 3 frameworks was even more informative. For example, we were able to identify that most problems were located in the *interface* and *information design* and were *effectiveness* problems (users being unable to complete the task) or *efficiency* problems (the level of difficulty in performing the task). Moreover, we were able to get a better idea of how to address the problems, knowing which heuristics they were not addressing, which were often the *match between the system and the real world* and *help and documentation*.

### Challenges and Limitations

This study has some limitations. First, scenarios were created by the research team and imposed on the participants. This may have affected the results, as the focus was on the task and might have hindered access to other useful information. Second, the data were analyzed by only 1 expert or researcher. The results were presented to the research team, who agreed on the big picture without determining proper intercoder agreement. Third, programming errors interfered with some tasks, which may have led to the loss of useful information on usability. These problems were identified as programming errors, but the participants were not able to perform the task. If it had been possible to perform the task, other usability problems might have been identified for the task. During the analysis, we also realized that there was a possible mapping issue between the different categories of the frameworks. For example, “The participant did not see the phone number” could have been identified as either *effectiveness* or *efficiency*. The interpretation relies on what the analyst was focusing on. It could be coded as *effectiveness* if we consider that the user needs to call the phone number, and it could be coded as *efficiency* if the phone number is one method (among several) to access information. Thus, the combination of the 3 conceptual frameworks does not bring a mutually exclusive categorization, but it reduces the risk of blind spots. Regardless of the category, we were still able to identify that the phone number was not perceived and needed more emphasis.

Other limitations were related to the study participants. First, the participants had a high level of education, as most had a university degree (n=8). Although this might represent the population of service providers, it is not representative of the caregiver population. In Québec, only 27.6% of caregivers had a university degree [45]. The second limitation was the age gap between the *offering resources* group and the *finding resources* group. Age is a determining factor in the use of internet products [7]. However, the *finding resources* group was mostly caregivers of functionally dependent older persons. Statistics indicate that these caregivers are often older themselves [5], which could explain the gap. The third limitation was the number of participants. Although we had 10 users, all of them were not testing exactly the same pages. Five users were testing each objective of the website. Nevertheless, looking at the results, we still consider that most of the problems seemed to emerge during this usability and UX assessment, without an absolute confirmation on the saturation of problems. As this study was exploratory and targeted an early version of the website, we are confident that we have collected sufficient information to improve the prototype.

Our results are transferable to a very limited extent to other eHealth systems. They are indeed related to a specific interactive product (website) dedicated to specific users (caregivers and service providers). Nevertheless, our analysis proposition combining the ISO [13], Nielsen heuristic [16], and elements of UX [20] is highly applicable to the usability or UX evaluation of other eHealth systems.

### Conclusions

This study provided improvement possibilities for a prototype co-designed with caregivers and service providers. We were able to identify several usability and UX problems. The 3 frameworks used for the analysis allowed us to understand the nature of the problem (ISO) [13] and the dimension where it lies (elements of UX) [20], as well as provide potential problem-solving solutions based on the predicted judgmental operations (Nielsen heuristics) [16]. Thus, we will continue the co-design process to address those problems by recruiting service providers and caregivers to co-design a new version of the prototype. Our analytical method, based on the 3 conceptual frameworks and their combination, broadened the perspective of the problems encountered. This combination of frameworks for usability and UX analysis is a novel contribution that is transferable to other eHealth systems, which contributes to the advancement of knowledge in the eHealth community.

## Appendix

Multimedia Appendix 1 Synthesis of results combining the 3 analysis frameworks.

## Abbreviations

5ST: 5-second test
ISO: International Organization for Standardization
UX: user experience
