# Supplementary material for: Usability of a Co-designed eHealth Prototype for Caregivers: Combination Study of Three Frameworks
Source: JMIR Hum Factors. 2021 Aug 18;8(3):e26532. doi: 10.2196/26532 (PMC8411328; doi:10.2196/26532)
Supplement: Multimedia Appendix 1 [file humanfactors_v8i3e26532_app1.pdf]

Multimedia Appendix 1. Synthesis of results combining the 3 analysis frameworks.

User experience element	Context of use		Effectiveness		Efficiency		Satisfaction	
	Problems, n (%)	Total, N	Problems, n (%)	Total, N	Problems, n (%)	Total, N	Problems, n (%)	Total, N
**Skeleton: interface design**	0 (0)	151	*22 (14.6)*[a]	*151*	*24 (15.9)*	*151*	*8 (5.3)*	*151*
Help and documentation	0 (0)	0	1 (4.5)	22	4 (16.7)	24	0 (0)	8
Consistency and standards	0 (0)	0	0 (0)	22	2 (8.3)	24	0 (0)	8
User control and freedom	0 (0)	0	4 (18.2)	22	6 (25)	24	2 (25)	8
Match between the system and the real world	0 (0)	0	2 (9.1)	22	2 (8.3)	24	1 (12.5)	8
Esthetic and minimalist design	0 (0)	0	0 (0)	22	3 (12.5)	24	0 (0)	8

	Flexibility and efficiency of use	0 (0)	0	0 (0)	22	1 (4.2)	24	0 (0)	8
	Help users recognize, diagnose, and recover from errors	0 (0)	0	0 (0)	22	0 (0)	24	1 (12.5)	8
	Error prevention	0 (0)	0	10 (45.5)	22	3 (12.5)	24	0 (0)	8
	Recognition rather than recall	0 (0)	0	4 (18.2)	22	2 (8.3)	24	1 (12.5)	8
	Visibility of system status	0 (0)	0	1 (4.5)	22	1 (4.2)	24	3 (37.5)	8
**Skeleton: information design**		*2 (1.3)*	*151*	*19 (12.6)*	*151*	*25 (16.6)*	*151*	*4 (2.6)*	*151*
	Help and documentation	0 (0)	2	8 (42.1)	19	7 (28)	25	1 (25)	4
	User control and freedom	0 (0)	2	0 (0)	19	0 (0)	25	1 (25)	4
	Match between the system and the real world	1 (50)	2	5 (26.3)	19	14 (56)	25	0 (0)	4

Help users recognize, diagnose, and recover from errors	0 (0)	2	3 (15.8)	19	0 (0)	25	0 (0)	4
Error prevention	1 (50)	2	3 (15.8)	19	1 (4)	25	0 (0)	4
Recognition rather than recall	0 (0)	2	0 (0)	19	3 (12)	25	1 (25)	4
Visibility of system status	0 (0)	2	0 (0)	19	0 (0)	25	1 (25)	4
**Structure: interaction design**	*0 (0)*	*151*	*4 (2.6)*	*151*	*11 (7.3)*	*151*	*4 (2.6)*	*151*
Help and documentation	0 (0)	0	0 (0)	4	2 (18.2)	11	0 (0)	4
User control and freedom	0 (0)	0	2 (50)	4	2 (18.2)	11	2 (50)	4
Match between the system and the real world	0 (0)	0	1 (25)	4	0 (0)	11	0 (0)	4
Esthetic and minimalist design	0 (0)	0	0 (0)	4	3 (27.3)	11	0 (0)	4

	Flexibility and efficiency of use	0 (0)	0	0 (0)	4	0 (0)	11	1 (25)	4
	Error prevention	0 (0)	0	0 (0)	4	2 (18.2)	11	0 (0)	4
	Recognition rather than recall	0 (0)	0	1 (25)	4	2 (18.2)	11	0 (0)	4
	Visibility of system status	0 (0)	0	0 (0)	4	0 (0)	11	1 (25)	4
**Skeleton: navigation design**		0 (0)	151	*2 (1.3)*	*151*	*6 (4)*	*151*	*1 (0.7)*	*151*
	Help and documentation	0 (0)	0	2 (100)	**2**	0 (0)	6	0 (0)	1
	User control and freedom	0 (0)	0	0 (0)	2	2 (33.3)	6	1 (100)	1
	Recognition rather than recall	0 (0)	0	0 (0)	2	3 (50)	**6**	0 (0)	1
	Visibility of system status	0 (0)	0	0 (0)	2	1 (16.7)	6	0 (0)	1
**Surface: sensory design**		*1 (0.7)*	*151*	*2 (1.3)*	*151*	*2 (1.3)*	*151*	*0 (0)*	*151*

	Match between the system and the real world	1 (100)	1	0 (0)	2	2 (100)	2	0 (0)	0
	Error prevention	0 (0)	1	1 (50)	2	0 (0)	2	0 (0)	0
	Recognition rather than recall	0 (0)	1	1 (50)	2	0 (0)	2	0 (0)	0
**Strategy: user needs**		*1 (0.7)*	*151*	*0 (0)*	2	*0 (0)*	2	*0 (0)*	0
	Match between the system and the real world	1 (100)	1	0 (0)	2	0 (0)	2	0 (0)	0

[a]Italicization denotes sum of subcategories.
